# Dopamine Concentration Changes Associated with the Retrodialysis of Methylone and 3,4-Methylenedioxypyrovalerone (MDPV) into the Caudate Putamen

**DOI:** 10.3390/brainsci14030265

**Published:** 2024-03-08

**Authors:** Robert Goldsmith, Amal Aburahma, Jon E. Sprague

**Affiliations:** The Ohio Attorney General’s Center for the Future of Forensic Science, Bowling Green State University, Bowling Green, OH 43403, USA; goldrob@umich.edu (R.G.); amal.aburahma@ttuhsc.edu (A.A.)

**Keywords:** dopamine, MDPV, methylone, cathinone, microdialysis, bath salts

## Abstract

Structural modifications to synthetic psychoactive cathinones (SPCs), a class of drugs that contain a β-keto modification of the phenethylamine pharmacophore of amphetamine, induce differences in dopamine transporter (DAT) activity. Here, in vivo retrodialysis was utilized to deliver the SPCs 3,4-methylenedioxypyrovalerone (MDPV, a DAT inhibitor) or methylone (a DAT substrate) into the caudate putamen of male Sprague-Dawley rats. Dialysate samples were collected prior to and post drug administration, and temporal changes in dopamine concentration were quantified using HPLC-EC methods. Methylone elicited a 200% increase and MDPV a 470% increase in dopamine levels at the 10 min time point. The findings demonstrate that in vivo retrodialysis can be used to evaluate the effects of SPCs on neurotransmission in the brain.

## 1. Introduction

**Figure 1:**
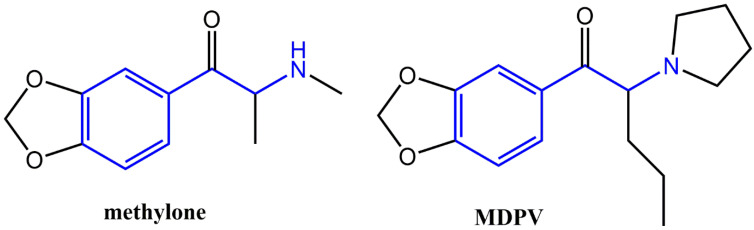
Chemical structures of methylone and MDPV. The phenethylamine portion of the molecule is indicated in blue.

The core scaffold of the pharmacophore of synthetic psychoactive cathinones (SPCs) is the β-keto-substituted phenethylamine or cathinone [1]. Cathinone is a naturally occurring alkaloid found in the leaves of the Khat plant Catha edulis that is indigenous to northeast Africa and the Arabian Peninsula [1]. Clandestine labs first began to synthesize synthetic cathinones in an attempt to circumvent laws associated with other popular sympathomimetic agents such as amphetamines and 3,4-methylenedioxymethamphetamine (MDMA) [2,3]. For the last decade, the introduction of newly synthesized psychoactive drugs including synthetic cathinones have been on the rise [2]. From a structural standpoint, the presence of the β-ketone increases the chemical polarity of the synthetic cathinones relative to their non-keto substituted phenethylamine analogues, although they still cross the blood–brain barrier [2,3]. Like other phenethylamines, these compounds can exist between two stereoisomeric forms with differing potencies [4] and can commonly be found as racemic mixtures [5]. Functional group differences in loci positions result in characteristic changes in how each compound modulates neural activity, primarily the dopamine transporter (DAT). The potency and binding affinity of the synthetic cathinones to DAT are influenced not only by the β-ketone substitution but also substitutions to the N-terminus and the α-position, as well as those found on the phenyl ring. Common substitutions at the N-terminus such as non-polar methyl/ethyl carbon chains aim to increase the lipophilicity of the compounds, as can be seen in methylone (Figure 1). Increased lipophilicity can also be achieved by the addition of electron-withdrawing groups [1]. Pyrrolidine derivatives such as 3,4-methylenedioxypyrovalerone (MDPV) are highly lipophilic due to the presence of less-polar, carbon-dense ring structure [6]. Substitutions at the α-position also commonly display differing carbon chain lengths, with methylone displaying only a methyl group while MDPV contains a propyl carbon chain (Figure 1). Phenyl ring modifications typically involve methyl groups at varying positions or a dioxolane ring, as found in methylone and MDPV. MDPV and methylone differ from each other by terminal amine and α-carbon substitutions. These chemical modifications result in MDPV functioning as a DAT blocker [7,8,9,10,11] and methylone as a DAT substrate, increasing dopamine release [7,12,13]. DAT blockers increase extracellular dopamine levels through their inhibition of dopamine reuptake, whereas DAT substrates are transported into the presynaptic terminal and reverse DAT activity to facilitate dopamine efflux into the extracellular space [14]. Moreover, recent studies have suggested that the relative selectivity of a compound to increase extracellular dopamine (DA) compared to serotonin (5-HT) levels was important for producing DA increases in the nucleus accumbens and reward [14,15,16]. For example, the addition of a 3,4-methylenedioxy group to methcathinone (methylone) resulted in a >100-fold decrease in selectivity for releasing DA vs. 5-HT [13,14,15,16,17,18]. In contrast to the literature cited above regarding the structure–activity relationship of amphetamine-like monoamine releasers, monoamine uptake inhibitor (MDPV)’s neurochemical effects on DA levels appear to be less susceptible to increases in 5-HT selectivity. For example, a recent and direct comparison of MDPV and methylone demonstrated that MDPV selectively increased DA levels more so than methylone [13,16]. Zwartsen et al. [19] investigated the differences between several synthetic cathinones in their acute effects on human dopamine, norepinephrine, and serotonin reuptake transporters (hDATs, hNETs, and hSERTs) that were transfected in human embryonic kidney (HEK) 293-cultured cells. In this study, methylone inhibited hDATs and hNETs with similar potency, whereas MDPV was a more potent inhibitor of both hDATs and hNETs [19]. From a structure–activity relationship standpoint, the study concluded that the presence of either a pyrrolidine moiety or a long alkyl α-carbon chain (as seen in MDPV) increased potency to inhibit hDAT compared to methylone. The inhibition of uptake via hNETs was associated with the presence of a pyrrolidine moiety. The study also examined the effects of methylone and MDPV on neuronal activity after acute (30 min) and prolonged (4.5 h) exposure compared to other psychoactive agents. Here, the addition of the α-ketone group in methylone compared to MDMA resulted in much lower neuronal activity after acute and prolonged exposure. On the other hand, MDPV resulted in a two- to fourfold increase in neuronal activity and neurotoxicity after acute and prolonged exposure compared to agents that lack the long alkyl α-carbon chain [19].

Furthermore, there is a lack of structure–activity relationship studies determining the in vivo neurochemical effects of mixed releasers/uptake inhibitors such as methylone. While previous studies have demonstrated the effects of peripheral doses of MDPV and methylone on DA extracellular concentrations in specific brain regions [8,15,16], no studies have yet been designed to examine the effects of the direct perfusion (retrodialysis) of these compounds into the brain.

Retrodialysis allows for a drug to be infused through a microdialysis cannula into a specific brain region without further perturbation of the surrounding tissue with subsequent injections [20]. In recent years, the retrodialysis technique has grown in popularity as a targeted drug delivery method with many applications in research [21]. Proctor et al. [21] showed that a newly synthesized ionic hydrogel membrane was capable of the controlled diffusion of DA via retrodialysis in a model system composed of two wells (one containing an aqueous solution of dopamine hydrochloride and the other containing a phosphate-buffered saline (PBS) solution) connected together with the ionic hydrogel membrane functioning as a bridge [21]. Interestingly, microdialysis and retrodialysis techniques are not solely used in in vivo systems or to assess catecholamine levels following drug administration [22,23,24]. In fact, these techniques were utilized successfully in numerous applications including, but not limited to, alterations in K+ concentrations in the perfusate to evoke dopamine release [22], testing biomarkers in the skin [23], and use in cell culture systems [24]. Matto et al. [22] aimed to explore the effect of the retrodialysis of a 100 mM [K+]-rich modified Ringer solution on striatal dopamine levels of wild-type, heterozygous, and homozygous Wfs1 null-mutant mice. The study concluded that the homozygous Wfs1 null-mutant mice showed significantly lower dopamine output compared to wild-type or heterozygous Wfs1 mutant mice after the direct perfusion of the high-concentration [K+] challenge into the mouse striatum [22]. The differences in dopamine levels evoked in each mouse group upon the [K+] challenge suggest that [K+] retrodialysis was carried out successfully. Baumann et al. [23] used a skin reservoir model to assess the validity of microdialysis as a sampling technique for the recovery of large biomarkers from previously frozen human skin specimens. Out of the 17 biomarkers under investigation, the relative recovery of 13 biomarkers was successfully specified in the range of 4.0–18.4%. Additionally, the research group noted that the fluid recovery using microdialysis sampling technique was stable, and no probe leakage during the experiments was observed [23]. Finally, Cheng et al. [24] utilized microdialysis probes for in vitro sampling to measure the levels of catecholamines in a pheochromocytoma cell culture medium that was enclosed in a Petri dish. In this study, the dialysis membrane was immersed in a culture medium containing the following catecholamines: norepinephrine, epinephrine, 3,4-dihydroxyphenylacetic acid (DOPAC), and DA. A liquid chromatography analysis of the dialysates detected all four catecholamines, suggesting that the microdialysis technique was successful at collecting catecholamines from the in vitro culture medium [24]. The results obtained from those studies further emphasize that microdialysis and retrodialysis techniques can be employed for a wide range of applications both in vitro and in vivo. In the present study, we used the in vivo retrodialysis of MDPV and methylone into the caudate putamen to analyze temporal changes in extracellular DA concentrations under the influence of structurally different synthetic cathinones.

## 2. Materials and Methods

### 2.1. Animals

Adult male Sprague-Dawley (Rattus norvegicus domesticus) (n = 10; 330 ± 28 g) rats were obtained from Envigo (Indianapolis, IN, USA). The animals were housed two per cage (cage size: 21.0 × 41.9 × 20.3 cm^3^) at 21–22 °C, maintained on a 12:12 h light/dark schedule, and provided ad libitum access to food and water. Animal maintenance and research were conducted in accordance with the eighth edition of the Guide for the Care and Use of Laboratory Animals as adopted and promulgated by the National Institutes of Health, and protocols were approved by the Bowling Green State University Animal Care and Use Committee.

### 2.2. Drugs and Chemicals

Dopamine (DA), 3,4-dihydroxyphenylacetic acid (DOPAC), and ketamine/xylazine were obtained as hydrochloride salts from Sigma-Aldrich Chemicals (St. Louis, MO, USA). MDPV and methylone were obtained from Cayman Chemicals (Ann Arbor, MI, USA) as hydrochloride salts. HPLC-grade methanol, phosphate hydrogen monobasic, octasulfonic acid, and a citric acid buffer were obtained from Sigma-Aldrich Chemicals (St. Louis, MO, USA). Artificial cerebrospinal fluid (147 mmol/L NaCl, 2.7 mmol/L KCl, 1.2 mmol/L CaCl_2_, 0.85 mmol/L MgCl_2_) was obtained from Hammarby Fabriksväg 43 (Stockholm, Sweden).

### 2.3. In Vitro Probe Recovery Evaluation

Prior to starting each in vivo experiment, the percent recovery of the microdialysis probe was evaluated in vitro. Briefly, the probe was emersed in 37 °C artificial cerebrospinal fluid (aCSF) for 15 min. Then the aCSF was replaced with a DA standard solution prepared in aCSF, and 3 recovery samples were collected at 5 min intervals and at a flow rate of 1.1 μL/min. The DA standard solution and the recovery samples were then assessed using HPLC-EC. For MDPV and methylone % recovery assessments, the drug under investigation (MDPV or methylone) was dissolved in aCSF and pumped through a microdialysis probe that was immersed in aCSF for 15 min at 37 °C at a 1.1 μL/min flow rate. After this calibration period, 3 dialysate samples were collected at 5 min intervals. Both the standard drug solutions and the dialysate samples were assessed using HPLC-EC. Finally, the % recoveries of DA, MDPV, and methylone were calculated using the following equation:

$$\%\;\mathrm{recovery}=\frac{AUCc\;of\;recovery\;sample}{\begin{array}{c} AUCc\;of\;standard\;sample \end{array}}\times 100\%$$

### 2.4. Surgical Cannula Placement and Retrodialysis

**Figure 2:**
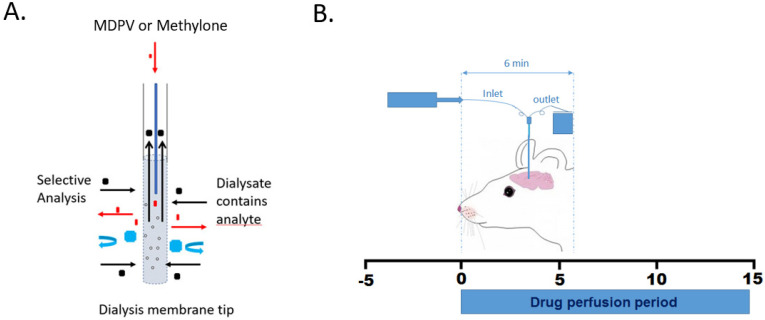
Study design. (**A**) The microdialysis probe is capable of (1) being surgically implanted into a specific brain region; (2) delivering chemical stimulus (red), such as methylone and MDPV, and selectively measuring the analyte (black), such as dopamine, by using different types of dialysis membrane tips and rejecting non-targeted chemicals (blue). (**B**) The timeline for synthetic cathinone perfusion. Time zero indicates the beginning of the synthetic cathinone perfusion. After the surgical implantation of the microdialysis cannula, the cannula was allowed to equilibrate for 90 min before three baseline samples were collected and the perfusion medium was changed to aCSF containing methylone or MDPV (100 mM in aCSF). After the 15 min perfusion period, the perfusion medium was converted back to aCSF for the remaining collection period.

For each trial, one rat was anesthetized with a 150 mg/kg dose of an 80 mg/mL ketamine/12 mg/mL xylazine solution, and upon complete sedation, placed onto a small, heated metal water bath inside a stereotaxic frame. To maintain sedation throughout the surgery, an intraperitoneal injection (0.15 mL) of a ketamine/xylazine mixture was administered on an as-needed basis. Each rat received supplemental oxygen at 1.5 L/min from a stereotaxic nosecone, and a rectal probe thermometer was inserted to monitor the rat’s core body temperature. An infrared oxygen sensor/heartbeat monitor was clipped to the right foot to monitor percent blood oxygen and heart rate during the surgery. The cannula was placed into the caudate putamen at the stereotaxic coordinates (AP = −0.48 mm; L = 3.4 mm; V = 6.0 mm), according to Paxinos and Watson [25]. For method development, the caudate putamen was selected to provide a large, dopamine-rich target. A microdialysis cannula (Harvard Apparatus, Holliston, MA, USA) with a 2.0 mm membrane length and a 20 kDa cutoff was connected to a microinfusion pump calibrated to deliver artificial cerebral spinal fluid (aCSF) at a rate of 1.1 μL/min. After a 90 min calibration period, 5 μL baseline samples were collected in 5 min intervals. The animals were treated with 100 mM of methylone (n = 5) or MDPV (n = 5) in the aCSF by direct retrodialysis while under ketamine/xylazine anesthesia at a rate of 1.1 μL/min through the microdialysis cannula for 15 min (Figure 2); dialysates were collected at 5 min intervals for 60 min post-treatment. After a series of dose–response studies and based on our previous findings of methylone concentrations in the brain of greater than 12,000 μg/L following a peripheral subcutaneous dose of 20 mg/kg [16], a 100 mM retrodialysis doses of MDPV and methylone were used. Paxinos and Watson [25] were referred to verify cannula placement following each study.

### 2.5. High-Performance Liquid Chromatography–Electrochemical Detection (HPLC-EC)

The mobile phase consisted of 12% methanol and an 88% mixture of 0.05 M phosphate, 0.03 M citric acid buffer, 0.6 mM octasulfonic acid, and 1.0 mM EDTA–disodium. The pump flow rate was 0.55 mL/min, and it was set to an operating temperature of 30 °C. Dopamine was separated using a PP-ODS II reverse-phase C18-column (Shimadzu, Colombia, MD, USA) and identified according to the retention time of the standard, and concentrations were quantified by comparison with the peak heights of the standard concentration curve (10^4^–10^8^ pg/μL). Standard concentration curves were determined before each microdialysis experiment to ensure the accuracy of standard retention times. The quantification of sample DA concentrations was performed using an Epsilon electrochemical (EC) detector connected to the HPLC device (Shimadzu, Canby, OR, USA). The detector’s sensitivity was 5 μA, and the oxidation potential was fixed at +700 mV using a glassy carbon working electrode versus an Ag/AgCl reference electrode. Lab Solution software (version 5.93) was used to integrate and analyze the raw data for the determination of DA and DOPAC levels.

### 2.6. Statistics

GraphPad InStat v.6.0 software was used to complete all statistical analyses of data. The results are presented as the mean ± SEM of the extracellular dopamine concentration of the treatment groups, expressed as a percentage of corresponding controls for ease of comparison. Within the time course of a treatment group, statistical significance was determined using an ANOVA with Dunnett’s post hoc test, carrying out a comparison back to time zero. Between-treatment-group differences were compared using an ANOVA followed by the Student–Newman–Keuls multiple-comparison test. Significance was established at *p* < 0.05 a priori.

## 3. Results

**Figure 3:**
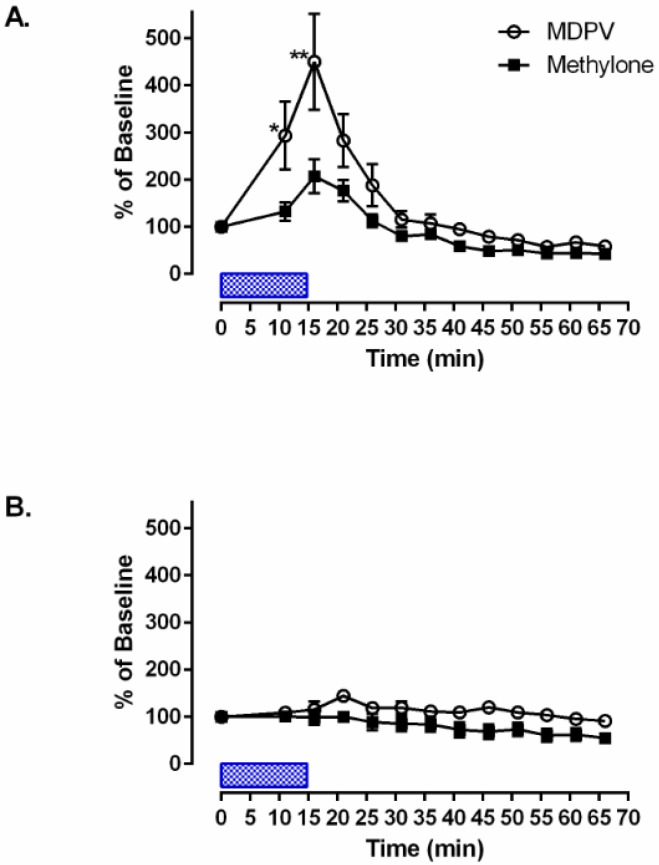
(**A**) Experimental extracellular dopamine concentration versus time plots for methylone and MDPV (100 mM) after direct infusion into the caudate putamen for 15 min (one-way ANOVA with a Student–Newman–Keuls post hoc test: F_(25,104)_ = 10.413, *p* = 0.0001). (**B**) Experimental extracellular DOPAC concentration versus time plots for methylone and MDPV (100 mM) after direct infusion into the caudate putamen for 15 min. Each value is the mean ± SEM. (n = 5). * indicates significant differences between treatment groups. A single * represents *p* = 0.01 and ** represents *p* = 0.001. Average baseline levels of dopamine were 805.3 ± 16.9 pg/uL.

Methylone and MDPV (100 nM) both significantly increased DA levels in the caudate putamen. DA levels began to increase immediately upon the drug reaching the target brain area and peaked 10 min after for both methylone and MDPV. Methylone elicited a 200% increase in baseline dopamine levels at the 10 min time point (Figure 3; one-way ANOVA with Dunnet’s post hoc test to baseline: F_(12,52)_ = 14.199, *p* = 0.01). MDPV increased DA levels by 470% compared to the baseline at the 10 min time point (Figure 3A; one-way ANOVA with Dunnet’s post hoc test to baseline: F_(12,52)_ = 9.208, *p* = 0.01). A significant difference was found between MDPV and methylone DA concentrations at 5 and 10 min post treatment. (one-way ANOVA with the Student–Newman–Keuls post hoc test: F_(25,104)_ = 10.413; at 5 min, *p* = 0.01 and at 10 min, *p* = 0.001). DOPAC levels were also assessed for both groups, and no significant change was observed (Figure 3B).

The percent recovery from in vitro calculations for the DA standard ranged from 26–36%. MDPV and methylone had percent recovery values ranging from 22–24%.

## 4. Discussion

By measuring the temporal changes in DA concentrations in vivo under the influence of two structurally different synthetic cathinones, we sought to determine differences in DA levels following the direct in vivo administration (through retrodialysis) of the DAT inhibitor MDPV and the DAT substrate methylone [7,13]. Receptor-binding profiles that measure agent-specific affinity for the DAT transporter indicate results consistent with our own, and in vitro work has determined MDPV to behave solely as a DAT blocker and possess both the highest affinity for DAT (Ki = 0.01 ± 0.002 μM) and the highest DAT monoamine transporter inhibition (IC_50_ = 0.031 μM) [4]. Methylone acts as a relatively less potent DAT reuptake blocker (IC_50_ = 4.82 μM) with a lower affinity for DAT (Ki = 2.73 ± 0.2 μM), but additionally acts as a dopamine releaser. Alongside DAT activity, methylone acts as a reuptake inhibitor for both SERTs and NETs. MDPV also displays NET reuptake inhibition at a two to four times lower potency than at DAT but does not block SERT reuptake [10]. Both the high affinity and selectivity of MDPV towards DAT (DAT/SERT ratio: >100) in comparison to the multi-transporter affinity of methylone (DAT/SERT ratio: 3.3) [10] are responsible for differences in each drug’s ability to increase synaptic DA.

MDPV and methylone both have a brain/plasma ratio much greater than 1 [3,26], which indicates that a compound freely crosses the blood–brain barrier [26,27]. Here, we demonstrate for the first time that the retrodialysis of methylone and MDPV results in DA concentration changes consistent with those seen with the peripheral administration of these agents. Similar to our present findings, Baumann et al. [13] measured a 1.7-fold increase in baseline DA concentrations within the nucleus accumbens following the peripheral administration of methylone (1 mg/kg, intravenous [iv]). Elmore et al. [28] saw a two-fold increase in dialysate concentration of DA in the nucleus accumbens compared to baseline following a 1 mg/kg iv dose of methylone. Upon the administration of two sequential iv injections (1.0 mg/kg followed by 3 mg/kg) of methylone, rats experienced increases of 200–300% above baseline dopamine concentrations in the nucleus accumbens [16]. Baumann et al. [8] conducted microdialysis studies with peripherally administered MDPV and demonstrated that 0.1/0.3 mg/kg iv injections of MDPV resulted in two-fold to four-fold increases, respectively, of basal DA in the nucleus accumbens. Schindler et al. [16] demonstrated that MDPV (0.1 mg/kg followed by 0.3 mg/kg, iv) exhibited a two- to four-fold increase when measuring extracellular DA concentrations in the nucleus accumbens. Recently, Johnson et al. [18] found a 300% increase in the DA concentration in the nucleus accumbens following MDPV administration (1 mg/kg, ip). In these studies, the peripheral administration of MDPV resulted in significantly higher increases in extracellular DA concentrations at lower doses than methylone. The DA release results in the present study suggest that MDPV likely possesses a higher risk for mediating reward than methylone due to its ability to increase synaptic DA to a significantly higher degree. Consistent with the present findings, Bonano et al. [29] examined the behavioral effects of four synthetic cathinones using intracranial self-stimulation and found that MDPV displayed a higher potency then methylone and a longer duration of action compared to the other compounds. Schindler et al. [16], using self-administration methods, demonstrated that the acquisition of a behavioral task was rapid under the influence of MDPV, whereas methylone was associated with a significantly slower acquisition period and comparatively lower response rates.

Previous studies have demonstrated that retrodialysis can be used to evaluate the pharmacokinetic and pharmacodynamic profiles of drugs [30,31,32,33]. For example, in vivo retrodialysis was utilized to study the effects of nociceptin (an endogenous neuropeptide) on cocaine-mediated increases in DA levels in the nucleus accumbens and on locomotor activity in freely moving rats [33]. Cocaine (20 mg/kg ip) resulted in an increase in extracellular DA levels that was suppressed by the administration of different concentrations of nociceptin (1 μM, 10 μM, and 1 mM) directly into the nucleus accumbens through a microdialysis probe [33]. The study further demonstrated that the co-retrodialysis of SB612111 (a selective nociceptin receptor antagonist) altered the nociceptin suppression of cocaine-enhanced DA release in the nucleus accumbens [33]. Nesbitt et al. [32] used retrodialysis to examine the protective effects of dexamethasone on neurochemical and histological disturbances induced by microdialysis probe implantation in the rat striatum [32]. Those authors showed that dexamethasone retrodialysis in the perfusion fluid provided protective effects against these disruptions in the 4–24 h post probe implantation [32]. Additionally, the elicited DA responses were stabilized by the localized perfusion of dexamethasone, as confirmed by fast-scan cyclic voltammetry measurements pre and post treatment [32]. Ferry et al. [30] explored the effect of the direct microinfusion of idazoxan (α_2_-AR antagonist, 0.1 mM) and UK 14,304 (α_2_-AR agonist, 10 μM) into the amygdala on norepinephrine levels before and after inhibitory avoidance learning [30]. The study reported that the retrodialysis of idazoxan consolidated norepinephrine release, while UK 14,304 diminished norepinephrine release, as confirmed by foot shock application [30]. Kozai et al. [31] utilized the retrodialysis method to study the influence of local dexamethasone perfusion into the cortex on modulating inflammatory responses resulting from probe insertion in brain tissue [31]. In this study, aCSF was directly perfused alone or with dexamethasone via microdialysis probes into transgenic mice that express a green fluorescent protein in brain microglia under the guidance of a CX_3_CR_1_ promoter [31]. In vivo two-photon microscopy imaging revealed that the microdialysis probe implantation caused acute morphological alterations in microglia that were reduced greatly during the T-stage morphology of the microglia and thus decreased the activation of these cells [31].

In a study by Hanberg et al. [34], retrodialysis was employed to determine the relative recovery of cefuroxime with and without the administration of a constant concentration of meropenem. Here, meropenem was chosen as an internal standard due to its similar physiochemical properties to cefuroxime. The successful recovery of cefuroxime via retrodialysis further supports the viability of using retrodialysis as an administration and recovery technique for chemical agents. Additionally, the research group also highlighted the challenge of decreased relative recovery associated with this technique and recommended that the RR should exceed 20% to minimize variations that arise due to low relative recovery (e.g., preanalytical handling, differences in chemical assays, low dialysate volumes, and low dialysate concentrations) [34]. Our findings with in vitro relative recovery studies for both methylone and MDPV were in line with the recommendation of the above-mentioned study.

In a similar study aimed at investigating the possibility of employing the retrodialysis technique as a calibration method, Yu et al. [35] performed a series of in vitro experiments that included both the microdialysis and retrodialysis of cefazoline with and without cefuroxime. Here, either normal saline was perfused (microdialysis) or cefazoline and/or cefuroxime were perfused (retrodialysis) at different concentrations and flow rates. The study concluded that the addition of cefuroxime to the microdialysis system of cefazoline did not affect the relative recovery of cefazoline. Furthermore, the relative recovery of perfusion solutions that contained both cefazoline and cefuroxime was similar to that of the dialysates obtained from the retrodialysis of either cefazoline or cefuroxime alone [35]. These studies provide a promising avenue for the possible use of the retrodialysis technique as a reliable and accurate method for the investigation of the pharmacological profiles of drugs including psychoactive agents.

A limitation of the present study is that the retrodialysis of a 100 nM concentration of MDPV or methylone was required to induce a change in dopamine concentration. Although previous studies have demonstrated that ketamine does not alter evoked dopamine release in anesthetized mice [36], the fact that we used a ketamine-anesthetized model may have contributed to the higher concentrations of MDPV and methylone required to increase dopamine levels. Further, this may seem like a “high dose”; our in vitro percent recoveries were 22–24% for MDPV and methylone. Further, the use of a microdialysis probe for drug delivery requires an equilibrium to be established between the two sides of the dialysis membrane.

## 5. Conclusions

Retrodialysis can be successfully utilized to deliver SPCs into specific brain regions while simultaneously using microdialysis to assess extracellular DA changes. Further research into the use of the retrodialysis of SPCs is necessary to enhance this overall methodology.

## Data Availability

Data are contained within the article.
